# Estimating long-term health risks after breast cancer radiotherapy: merging evidence from low and high doses

**DOI:** 10.1007/s00411-021-00924-8

**Published:** 2021-07-17

**Authors:** Cristoforo Simonetto, Daniel Wollschläger, Pavel Kundrát, Alexander Ulanowski, Janine Becker, Noemi Castelletti, Denise Güthlin, Elena Shemiakina, Markus Eidemüller

**Affiliations:** 1 Institute of Radiation Medicine, Helmholtz Zentrum München, Ingolstädter Landstraße 1, 85764 Neuherberg, Germany; 2 Institute of Medical Biostatistics, Epidemiology and Informatics, University Medical Center Mainz, Obere Zahlbacher Str. 69, 55131 Mainz, Germany; 3 Department of Radiation Dosimetry, Nuclear Physics Institute of the Czech Academy of Sciences, Na Truhlářce 39/64, 180 00 Prague 8, Czech Republic; 4 Present Address: IAEA Environment Laboratories, International Atomic Energy Agency, 2444 Seibersdorf, Austria; 5 Present Address: Division of Infectious Diseases and Tropical Medicine, University Hospital, Ludwig-Maximilians-Universität (LMU) Munich, 80802 Munich, Germany; 6 Present Address: Department of Radiation Protection and Health, Federal Office for Radiation Protection, Ingolstädter Landstraße 1, 85764 Neuherberg, Germany

**Keywords:** Radiation risk, Risk models, Breast cancer radiotherapy, Second primary cancer, Heart disease

## Abstract

In breast cancer radiotherapy, substantial radiation exposure of organs other than the treated breast cannot be avoided, potentially inducing second primary cancer or heart disease. While distant organs and large parts of nearby ones receive doses in the mGy–Gy range, small parts of the heart, lung and bone marrow often receive doses as high as 50 Gy. Contemporary treatment planning allows for considerable flexibility in the distribution of this exposure. To optimise treatment with regards to long-term health risks, evidence-based risk estimates are required for the entire broad range of exposures. Here, we thus propose an approach that combines data from medical and epidemiological studies with different exposure conditions. Approximating cancer induction as a local process, we estimate organ cancer risks by integrating organ-specific dose–response relationships over the organ dose distributions. For highly exposed organ parts, specific high-dose risk models based on studies with medical exposure are applied. For organs or their parts receiving relatively low doses, established dose–response models based on radiation-epidemiological data are used. Joining the models in the intermediate dose range leads to a combined, in general non-linear, dose response supported by data over the whole relevant dose range. For heart diseases, a linear model consistent with high- and low-dose studies is presented. The resulting estimates of long-term health risks are largely compatible with rate ratios observed in randomised breast cancer radiotherapy trials. The risk models have been implemented in a software tool PASSOS that estimates long-term risks for individual breast cancer patients.

**Supplementary Information:**

The online version contains supplementary material available at 10.1007/s00411-021-00924-8.

## Introduction

Breast cancer is the most frequent cancer in women (Bray et al. 2018). With improved early diagnosis and advanced treatment approaches, current survival is high with 5- and 15-year relative survival rates of 91% and 80% (Breast Cancer Facts and Figures 2019–2020), respectively. The treatment often includes radiotherapy (RT) which reduces the risk of local recurrence and improves survival (Early Breast Cancer Trialists’ Collaborative Group 2011).

Depending on tumour type, stage and other characteristics, diverse target concepts are used in breast cancer RT, including whole- as well as partial-breast irradiation. To varying extent, regional lymph nodes are included in the fields. Breast cancer RT can be applied by several techniques including tangential or multi-field irradiation, in prone or supine position, under free breathing or breath hold. Additional boost irradiation may be applied intraoperatively, by brachy- or teletherapy. All treatment strategies share the general aim of RT, namely to deliver a sufficiently high radiation dose to the target volume to eradicate the tumour and prevent recurrences. At the same time, the unwanted exposure of healthy tissues should be reduced to limit adverse treatment effects. However, although modern RT techniques allow for flexible dose distributions, nearby and to a lesser extent also distant healthy tissues still receive considerable radiation doses. This may lead to complications during the course of RT or within weeks or months. Moreover, these exposures can lead to the induction of heart disease or second primary cancer years afterwards, and the disease rates can remain elevated until the end of life. With increased cure rates and prolonged survival, these long-term health risks become increasingly relevant. Even though modern treatment techniques offer the possibility to reduce the exposure of the most critical organs at the expense of less critical ones, systematic and quantitative assessments of the long-term risks are lacking to date.

The major reason is the lack of reliable organ-specific risk models that can be applied to compare different dose distributions. While large parts of organs receive relatively low doses^1^ of a few Gy or even below 1 Gy, small parts of nearby organs are often exposed to dose levels comparable to the therapeutic dose delivered to the tumour, of the order of 50 Gy. For breast cancer RT, this refers in particular to the heart, ipsilateral lung, and bone marrow.

Risk at high doses has been investigated by a number of studies on radiation-induced long-term risk after RT, often performed as case–control studies (NCRP 2011). Due to the nature of such long-term effects, these studies rely on data from RT techniques from previous decades, whose dose distributions may considerably differ from current exposures. Thus, results from these studies may only be applied to modern RT with caution. Moreover, these risk estimates are most relevant for areas exposed to high doses. However, lower doses may considerably contribute to overall risk (Simonetto et al. 2019a). Biological responses differ between low and high doses (NCRP 2011, Sachs 2005), and therefore, radiation-induced health risks may be different. Best statistical evidence in the low-dose region is provided by radiation-epidemiological studies.

To address this issue, we propose to combine available risk estimates from both high- and low-dose regions to obtain organ-specific dose–response relationships over the full relevant dose range. Cancer induction is approximated as a local process, to which the whole organ is equally susceptible. With this assumption, organ cancer risk is obtained by integrating the dose–response relationship over the organ dose distribution. At high doses, the organ-specific dose response is taken from a meta-analysis of relevant medical studies. At low doses, the risk models are primarily taken from the Life Span Study (LSS) of the atomic bomb survivors of Hiroshima and Nagasaki. At intermediate doses, the risk models from the different dose regions are interpolated. The dose ranges are cancer-specific and are chosen for each endpoint based on the epidemiological evidence. Generally, the intermediate region corresponds to doses from about 0.5–1 Gy up to about 1–4 Gy.

The aim of the present study is to provide a framework for estimating long-term health risks for variable organ dose distributions over a wide dose range together with adjusted risk models and consideration of uncertainties. These risk models can be combined with individual-specific exposures to help guide RT treatment planning in a personalised way (Eidemüller et al. 2019). The risk models have been implemented in the dedicated software tool PASSOS^2^ (2021) for calculation of individual risks in a clinical setting.

## Methods

For cancer incidence, low- and high-dose data can substantially differ, not only in terms of the risk coefficients but also regarding the shape of the dose response. This work aims to integrate both pieces of information. Central to this approach is the assumption of locality of cancer induction by ionising radiation: it implies that cancer risk can be inferred from the dose at the local site that forms the origin of a tumour. Potential long-distance or systemic effects are assumed to be of minor importance. The absolute risk from different volume elements can then be added to yield the organ excess absolute rate (EAR). Analogously, dividing by the organ baseline risk, the organ excess relative risk (ERR) can be obtained by integration of the local excess relative risk, ${\text{ ERR}}_{l} \left( d \right)$, which describes the risk at a specific volume element *v* with dose *d*:

1 $${\text{ERR}} = \frac{{\mathop \smallint \nolimits_{V} {\text{ERR}}_{l} \left( {d\left( v \right)} \right){\text{d}}v}}{{\mathop \smallint \nolimits_{V} {\text{d}}v}} .$$

The volume integral is confined to the volume $V$ of the organ of interest, and $d\left( v \right)$ denotes the local dose in a small volume *v* within the organ. Here, ${\text{ERR}}_{l}$ is assumed to depend only on the local dose and potential variations within the organ in susceptibility to cancer induction are neglected. A similar concept was also used by Schneider and Walsh (2008) for definition of the organ equivalent dose. If the dose–response relationship is linear, ${\text{ERR}}_{l} \left( d \right) = {\text{ERR}}_{pd} \cdot {d}\left( v \right)$, Eq. (1) turns into ${\text{ERR}} = {\text{ERR}}_{pd} \cdot d_{{{\text{mean}}}}$, where ${\text{ERR}}_{pd}$ is the excess relative risk per dose and $d_{{{\text{mean}}}}$ is the mean organ dose.

**Fig. 1:**
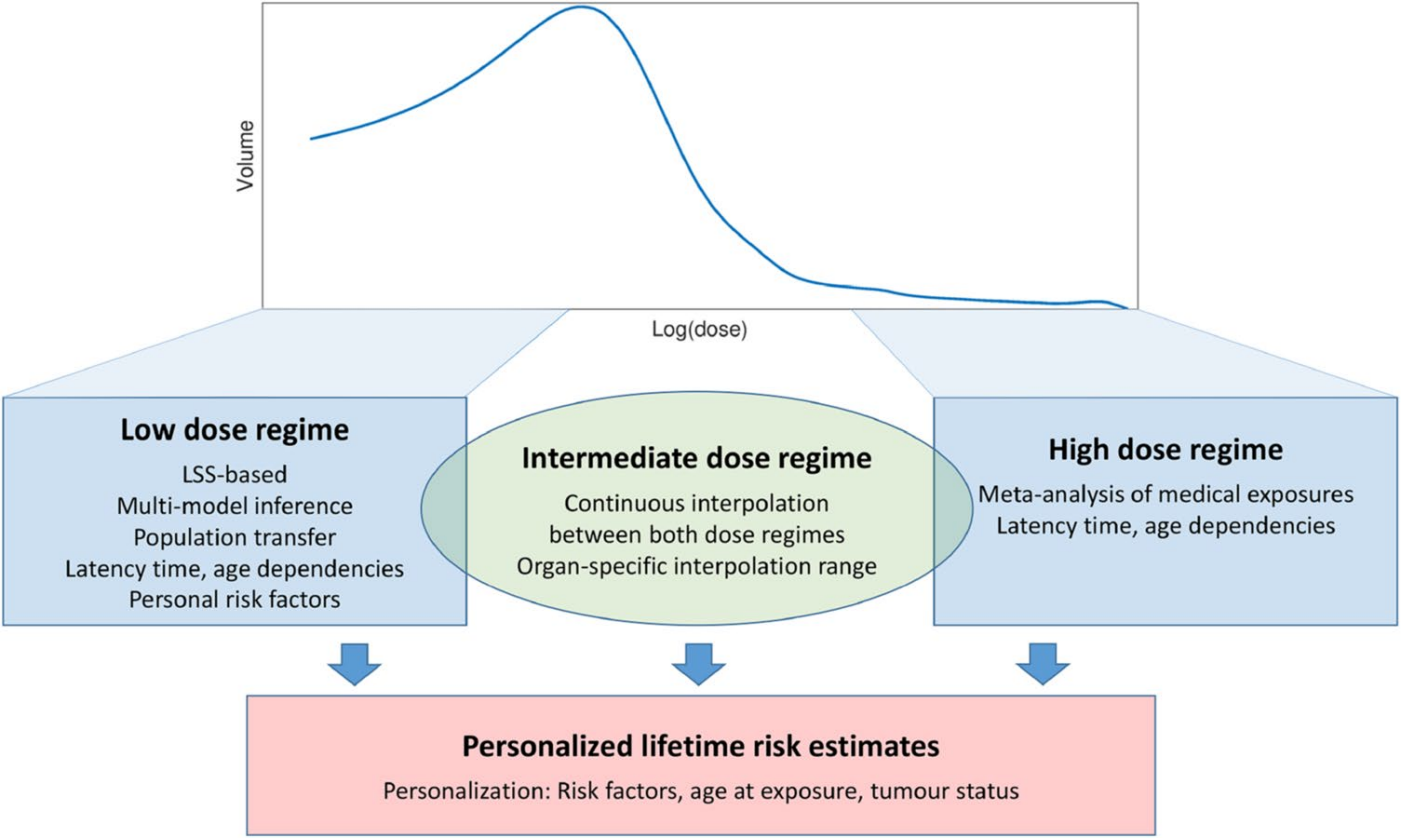
Schematic representation of the cancer risk model development. The graph sketches a possible organ dose distribution. Different models are applied to different dose regimes. The final combined risk models can be used to calculate personalised lifetime risk for variable dose distributions (colour figure online)

Our methodological approach is schematically represented in Fig. 1. A small part of the volume of nearby organs receives high doses. In this region, risk estimates from epidemiological studies after medical exposure are used, based on a meta-analysis of published studies for second primary cancer in the lung, contralateral breast, and for secondary leukaemia. A larger volume fraction in all nearby organs and essentially whole volumes of remote organs are exposed to low doses. Here, organ-specific risk models are mainly obtained from the LSS. In the intermediate dose region, defined organ-specifically based upon available epidemiological evidence, an interpolation between the low- and high-dose risk models is performed.

Virtually all organs were considered in the present work, as described in detail below. However, no attempt was made to estimate radiation-induced risks of skin, soft tissue and bone cancers or lymphoma. For these endpoints, data on risks are scarce over the full dose range, organ dose distributions are difficult to determine, and the absolute increase in cancer cases is moderate (Clarke et al. 2005; Taylor et al. 2017). Moreover, no attempt was made to estimate radiation-induced risk of second primary cancer in the treated breast, as the authors are not aware of any study on this subject.

### High-dose risk models

Some organ parts receive high doses during breast cancer RT since they are close to or even partially within the treatment fields. In this high-dose region, the risk was assessed by a meta-analysis of published studies on the dose response of heart disease, lung cancer, contralateral breast cancer and leukaemia after medical irradiation. The studies were identified by performing a PubMed search as well as by reference tracking using recent reviews and articles.

The meta-analysis only included studies for which a relative risk (RR) per dose category, or alternatively an excess relative risk (ERR) or excess odds ratio (EOR) and corresponding uncertainty intervals could be extracted or determined. Studies without exposures higher than 3 Gy were excluded. For repeated analyses on the same patient cohort, only the most recent study was retained.

Information from each publication was extracted to a spreadsheet, including the age group of exposed individuals, diseases treated with radiotherapy, the analysed endpoint, sample size, duration of follow-up, and statistical model with type of dose–response analysis.

For heart disease, lung cancer and breast cancer, linear risk models were obtained by the meta-analysis. For each study that did not report an estimate of linear excess relative risk per dose (ERR_*pd*_), but instead provided results for dose categories with associated relative risks, the following methods were used to obtain a linear ERR_*pd*_ estimate and its confidence intervals: the reference dose for each given dose category was set to the category mean or median dose, or to the mid-point of the interval when category means or medians were not reported (Doi et al. 2014). When the highest dose category had no upper boundary, its reference dose was set to the lower boundary plus the length of the second highest interval. A linear ERR_*pd*_ estimate was derived as the slope from an inverse-variance weighted linear regression of the relative risks on the category reference doses without intercept and an offset of 1 (Little 2001). The variance of a category’s relative risk estimate was calculated based on the width of its confidence interval assuming a log-normal distribution. The confidence interval for the ERR_*pd*_ estimate was calculated using parametric bootstrapping with 1000 replicates with the derived category variances assuming a log-normal distribution (Doi et al. 2014). Finally, the individual ERR_*pd*_ estimates from different studies were combined, using a random effects meta-analysis (Viechtbauer 2010). The meta-analysis was characterised using Cochran’s *Q*, number of degrees of freedom, *p* value for test of heterogeneity, and *I*^2^ measure for inconsistency (Higgins et al. 2003). Assuming a log-normal distribution of ERR_*pd*_ + 1, confidence intervals and standard deviations were derived. Therefore, the results of the meta-analysis are models linear in dose *d* parametrised by

2 $${\text{ERR}}_{l} \left( d \right) = {\text{ERR}}_{pd} \cdot d = \left( {e^{\beta } - 1} \right) \cdot d ,$$

where $\beta$ is sampled from a normal distribution, and ${\text{ERR}}_{l}$ denotes the local excess relative risk. For leukaemia, different studies were combined directly, as discussed below.

### Low-dose risk models

Most organs in the body receive only low doses during breast cancer RT. At low doses, the most informative study for risk inference is the LSS, which allows to derive organ-specific risk models and to analyse potential age dependencies. The LSS includes doses up to about 4 Gy. Radiation risk models for cancer were previously developed from the LSS within the ProZES project, which aimed to assess the probability that a given cancer was caused by a preceding radiation exposure (Ulanowski et al. 2020). The ProZES models were developed together with an international expert group and approved by the German Commission on Radiological Protection (Strahlenschutzkommission, SSK) and the German Federal Office for Radiation Protection (Bundesamt für Strahlenschutz, BfS). Furthermore, the models allow assessment of uncertainty from different sources. The models include organ-specific risk models for the most frequent solid cancer sites, in particular the lung, breast, stomach, colon and thyroid. For less frequent cancers, models were grouped for functionally similar organs. Therefore, with some adaptations discussed below, these models were also used in the current work to estimate risk of second primary cancer from low doses in breast cancer RT.

### Interpolation between low- and high-dose risk models

With the aim to construct a dose–response relationship for the full dose range from low to therapeutic doses, an intermediate dose range was defined in which the excess relative risks from the low- and high-dose models were linearly interpolated. At the lower boundary of this transition region, the excess relative risk corresponded to the low-dose model, and at the upper boundary to the high-dose model. The transition region was chosen on an organ-specific basis according to the availability of epidemiological information, as described in the Results section. If the risk from the low-dose model at the lower boundary of the transition region was higher than the risk from the high-dose model at the upper boundary, linear interpolation would result in a local minimum of the dose–response relationship. Local minima might be difficult to understand biologically and could have strong impact on optimisation of RT dose distributions with regard to risk. To avoid introducing such minima by the interpolation procedure, the geometric mean of the two risk values was taken and used as a constant risk over a correspondingly enlarged interpolation region.

### General aspects of risk assessment

Risk assessment involves a number of methodological issues. To a large extent, we adopted the approach developed in ProZES (Ulanowski et al. 2020) and thus discuss the concepts only shortly in the following.

#### Multi-model inference

To reduce the dependence of the risk estimates on the choice of one particular model and to provide a more realistic assessment of uncertainties, the low-dose models were built up of a superposition of different models, using multi-model inference with weights defined by the quality of fit of the models to the data. A similar approach was used for the high-dose model for leukaemia, as explained below.

#### Latency time

Radiation-induced cancer is only observed several years after the exposure since the induced cellular changes need time to develop into a tumour. This latency time between exposure and cancer is larger for solid cancer than for leukaemia. Risk was modelled to start after about 2 years after exposure and to increase until about 5 years for solid cancer, and 1 year and 2 years for leukaemia, respectively. After that time, cancer can be induced by the exposure without risk reduction (Ulanowski et al. 2020). This latency function was used in an identical way for the low- and high-dose risk models. For heart diseases, the parametrisation of latency was derived by analysing the literature directly in this work and is thus described in the Results section.

#### Risk transfer between populations

For the low-dose models, risk estimates are mainly based on the Japanese LSS cohort exposed in 1945 while this work aims to estimate risks for present-day German patients. Therefore, risk estimates have to be adapted (“transferred”) to account for population differences. Two common choices are multiplicative and additive risk transfer: multiplicative risk transfer assumes that ERRs are identical for the target population and the radiation-epidemiological cohort, while for additive risk transfer the same is assumed for EARs. To take the uncertainty due to the transfer into account, a stochastic mixture between both transfer types was implemented (Ulanowski et al. 2020).

For the high-dose models based on medical studies, only a purely multiplicative risk transfer was used, for several reasons. First, usually only ERR values (or excess odds ratios) without information on baseline rates were available. Second, the high-dose results were mostly derived from RT patients treated just a few decades ago, whose baseline cancer rates were likely substantially closer to those of current RT patients than was the case for the Japanese LSS population. In addition, multiplicative risk transfer was consistent with the assumption of compatible relative risk estimates in the meta-analyses.

#### Uncertainty evaluation

An important part of the present approach was a comprehensive assessment of related uncertainties. Following previously used methodology (Ulanowski et al. 2020), all uncertain input parameters were repeatedly sampled over their respective distributions by Monte Carlo methods. In particular, the following sources of uncertainty were explicitly taken into account: uncertainty in model selection; statistical uncertainty of a particular risk model, i.e. confidence intervals and correlations of model parameters; uncertainty in the interpolation dose range; uncertainty in cancer rates at young ages; uncertainty in the risk transfer; and uncertainty in the latency time.

#### Data base for risk estimates

Risk estimates are reported as the median with the 95% confidence interval. Cancer incidence rates were obtained from the German Centre for Cancer Registry Data (RKI 2013). For lung cancer, smoking intensity is an important risk factor, and hence it was explicitly taken into account using smoking-dependent lung cancer rates for the German population, see Supplementary Material. For heart diseases, mortality instead of incidence was estimated. Reasons included the wide range of severities for heart diseases, and the absence of a national register of heart disease incidence. Data were retrieved from German death statistics (Federal Health Reporting 2016). As breast cancer irradiation affects mainly the chest, only diseases with heart involvement were included (ICD-10 codes: I05-I52).

Example risk estimates using the presented framework were evaluated for different organs with a dose distribution derived from a standard left-sided whole breast 3D-conformal (3D-CRT) treatment plan for a woman with normal anatomy. The corresponding plan and dosimetry details can be found in References (Kundrát et al. 2019a; Simonetto et al. 2019a).

## Results

The risk models for lung cancer, contralateral breast cancer, leukaemia and heart disease are presented together with model-specific characteristics. For the different endpoints, first the high- and low-dose risk models are specified, followed by the interpolation scheme and the resulting dose–response relationship. Finally, risk estimates are presented for various sites for the considered 3D-CRT treatment plan and compared with rate ratios observed in randomised breast cancer RT trials.

### Lung cancer model

**Fig. 2:**
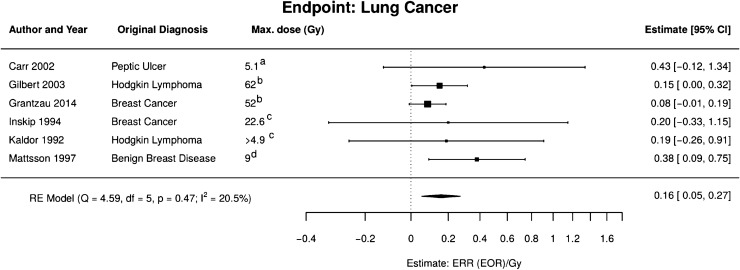
Meta-analysis of the high-dose studies for radiation-induced lung cancer. *RE model*  random effects model, *Q* Cochran’s *Q*, *df* degrees of freedom, *p* *p* value for test of heterogeneity; I^2^ measure for inconsistency, *ERR*  excess relative risk, *EOR* excess odds ratio. Dose definitions: a mean dose to the left lung; b dose to specific location of the secondary tumour; c mean dose to affected lung; d mean lung dose (colour figure online)

In the meta-analysis for lung cancer after moderate and high doses, two studies were included on irradiation of patients for Hodgkin lymphoma (Gilbert et al. 2003; Kaldor et al. 1992), one on peptic ulcer (Carr et al. 2002), one on benign breast disease (Mattson et al. 1997), and two studies on breast cancer patients (Grantzau et al. 2014; Inskip et al. 1994), see Fig. 2. A study on patients with tuberculosis (Howe 1995) was excluded since the lung disease or the non-radiation treatment might have influenced the risk of lung cancer. Moreover, a study on patients treated with X-rays for ankylosing spondylitis (Weiss et al. 1994) was excluded as patients were compared to the general population to estimate the risk. The result of the meta-analysis is an excess relative risk (ERR) model linear in dose $d$ with ${\text{ERR}}_{pd} = 0.16$ (95% CI 0.05, 0.27) Gy^−1^, see Eq. (2). The area of the box squares is proportional to the weight of the corresponding study in the meta-analysis.

For low doses, similar to Ulanowski et al. (2020), we based the structure of the models on the work by Furukawa et al. (2010), omitting parameters that were not statistically significant. Parameters for unknown smoking were introduced in the radiation response for both sexes as the ERR depends on smoking intensity: it peaks for about 5–10 cigarettes per day, and drops for higher smoking intensities (Furukawa et al. 2010; Cahoon et al. 2017). The models were fitted to the most recent LSS data (Cahoon et al. 2017) and combined by multi-model inference. Compared to the original analysis, the estimated lung cancer risk 10 years after irradiation increased by about 10% for a woman irradiated at age 50.

One of the studies importantly contributing to our meta-analysis (Grantzau et al. 2014) indicated an ERR below 0.26 for the range 1–4 Gy at the 95% confidence level. Depending on smoking intensity, this is in conflict with the low-dose model. Thus, low- and high-dose models are interpolated between 0.5 and 1 Gy.

**Fig. 3:**
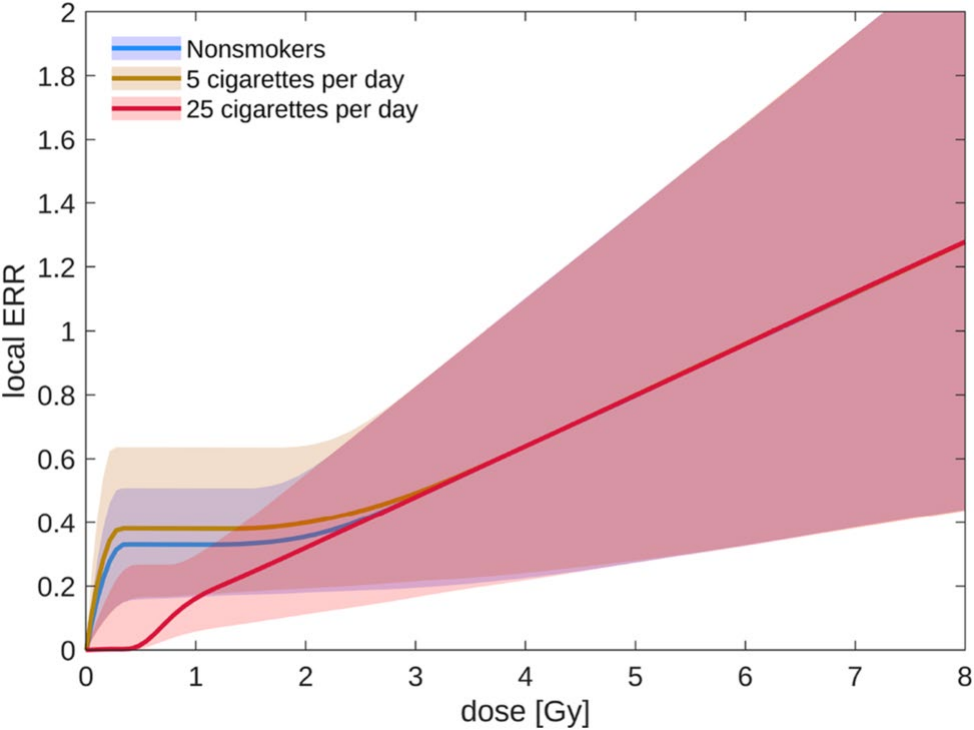
Lung cancer dose–response relationship for a 60-year-old woman irradiated at age 50, evaluated for different smoking intensities. Shaded regions correspond to 95% confidence intervals (colour figure online)

The resulting lung cancer dose response over the whole dose range is illustrated in Fig. 3 for a 60-year-old woman after RT at age 50 for different smoking intensities. At low doses, the risk increases sharply with increasing dose for low smoking intensities (blue and brown line). The high-dose model is not sensitive to smoking and predicts a less steep increase. Therefore, for low smoking intensities, the interpolation region was enlarged to permit linear interpolation without a local minimum. Smooth transitions between the different dose regimes originate from parameter sampling. For high smoking intensities (red line), the combined model is sublinear at low doses. The mean lung dose of our example dose distribution was 3.8 Gy. Due to the non-linearity, however, the mean dose is not sufficient to calculate the risk, but the organ cancer risk was obtained from integrating the local risk over the organ volume, Eq. (1). As a consequence, there was a moderate variation of the organ-integrated ERR with smoking intensity for the example dose distribution: from ERR = 0.80 for a 60-year-old woman who smoked 5 cigarettes per day down to ERR = 0.60 for a strong smoker with 25 cigarettes per day. On the other hand, EARs given per 10^5^ person years at age 60 strongly increased with smoking: from 20 for a non-smoker, 68 for a woman who has been smoking 5 cigarettes per day since age 20, to 356 for a woman who has been smoking 25 cigarettes instead. This largely enhanced EAR followed from the strong impact of smoking on the baseline risk.

### Contralateral breast cancer model

**Fig. 4:**
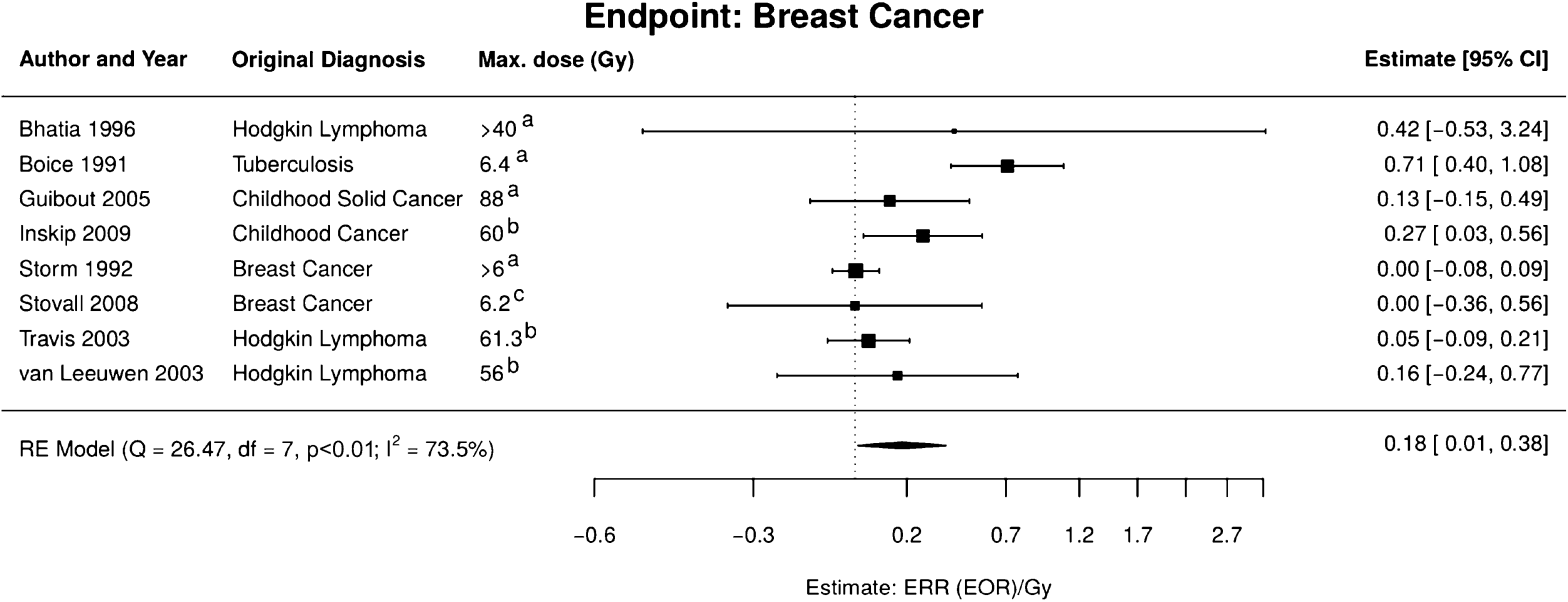
Meta-analysis of the high-dose models for radiation-induced breast cancer. *RE model* random effects model, *Q* Cochran’s *Q*, *df*  degrees of freedom, *p* *p* value for test of heterogeneity, I^2^ measure for inconsistency, *ERR*  excess relative risk,* EOR* excess odds ratio. Dose definitions: a mean breast dose; b dose to specific location of the secondary tumour; c dose to breast quadrant of the secondary tumour (colour figure online)

The meta-analysis of high-dose data (Fig. 4) included three studies on patients with Hodgkin lymphoma (Bhatia et al. 1996; Travis et al. 2003; van Leeuwen et al. 2003), one on tuberculosis (Boice et al. 1991), two on childhood cancer (Guibout et al. 2005; Inskip et al. 2009), and—most appropriate for our objective—two studies on contralateral breast cancer after breast cancer RT (Storm et al. 1992; Stovall et al. 2008). Studies on patients irradiated at infancy were not included as the breast undergoes substantial changes in childhood. Furthermore, also a study on radiation treatment of benign breast disease (Mattsson et al. 1993) was not taken into account to exclude any potential impact of the treated disease on later cancer risk. From the meta-analysis, an ${\text{ERR}}_{pd} = 0.18$ (95% CI 0.01, 0.38) Gy^−1^ was obtained, however, with substantial heterogeneity between the studies.

The low-dose model for breast cancer was directly adopted from Ulanowski et al. (2020). It is based on a pooled analysis of data from the LSS and several cohorts with medical radiation exposure (Preston et al. 2002). The risk model is an excess absolute rate model with explicit dependence on attained age and age at exposure. In particular, the relative risk decreases with increasing age at exposure.

The majority of the analysed high-dose studies do not allow estimating breast cancer risks below 1–2 Gy. In Travis et al. (2003) and Inskip et al. (2009), there is indication for increased risk at dose categories around 4 Gy, but only at higher doses risk is significant at the 95% confidence level. Storm et al. (1992) did not observe significant radiation risk even for the highest dose categories (between 2 and 3 Gy, and above 3 Gy with a mean of 4.6 Gy). Taken together, these figures do not contradict the risk estimates from the low-dose model, which is strongly driven by the LSS data with exposures up to 4 Gy. In view of these facts, the low- and high-dose risk models for the contralateral breast were linearly interpolated between 1 and 4 Gy in this work.

**Fig. 5:**
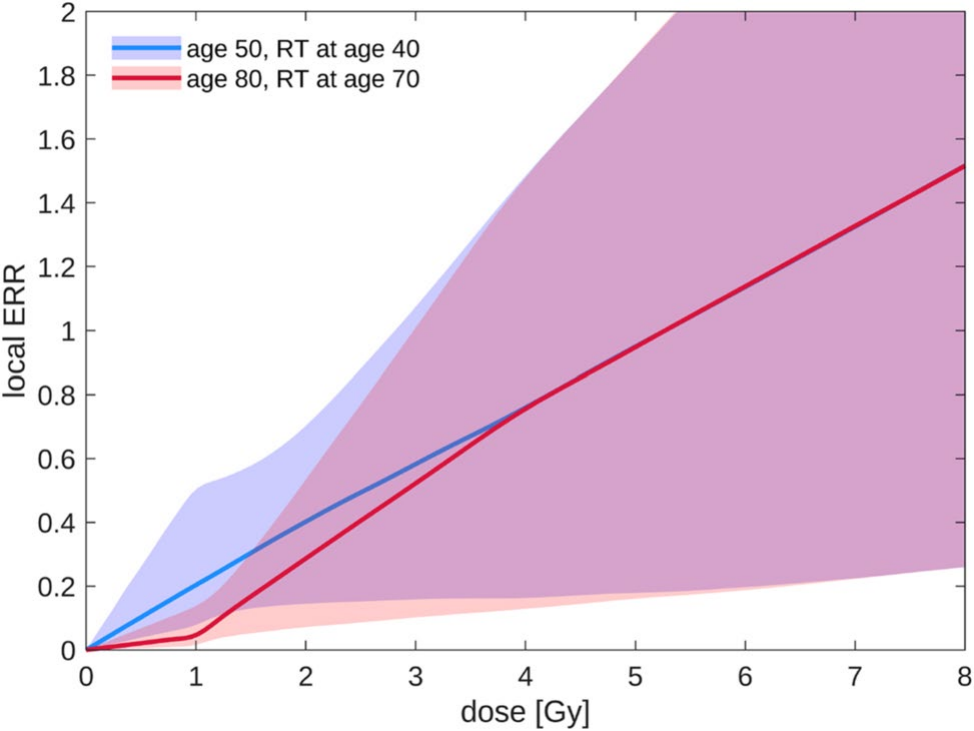
Dose–response relationship for radiation-induced breast cancer, evaluated for different ages. Shaded regions correspond to 95% confidence intervals (colour figure online)

The resulting dose–response curve of the combined risk model over the whole dose range is illustrated in Fig. 5 for 10 years after treatment at age 40 or 70. For the example 3D-CRT dose distribution with mean contralateral breast dose of 1.0 Gy, the organ-integrated ERR and EAR at age 50 are 0.19 and 42 per 10^5^ person years for a woman treated at age 40. For treatment at age 70, the ERR is 0.06 and the EAR is 24 per 10^5^ person years at age 80.

### Leukaemia model

Leukaemia is a group of blood cancers predominantly originating in the bone marrow. The active (red) bone marrow is distributed over many bones in the body and thus exhibits the strongest dose gradient in breast cancer RT. There is evidence in the literature that the shape of the dose–response relationship is non-linear (Blettner and Boice 1991; Curtis et al. 1994). However, only some studies on high doses have tested non-linearities and there is no generally accepted functional form of the dose–response relationship. Therefore, a meta-analysis was not feasible. Instead, we directly included models from four different studies. We did not include studies on leukaemia after childhood cancer and gave preference to studies which evaluated the existence of non-linearities in the dose–response. Results are summarised in Table 1. If several models were presented in a study, only the preferred model was retrieved. In Curtis et al. (1994) two different models were preferred depending on RT technique. Therefore, these two models have been assigned a weight of 12.5% while other models were sampled with a weight of 25%. Asymmetric confidence intervals were assumed to follow Eq. (2).

**Table 1:** Models contributing to the leukaemia high-dose model

Study	Model	Parameters	Comments
Blettner and Boice (1991)	$1 + {\text{ERR}}_{l} \left( d \right) = \left[ {1 + 0.1 \beta_{1} d} \right] \exp \left( { - \,0.1 \beta_{2} d} \right)$	$\beta_{1} = 8.8 \,\left( {95\% \,{\text{CI}} \,1.9, 31} \right) \,{\text{Gy}}^{ - 1}$ $\beta_{2} = 0.8 \,\left( {95\% {\text{ CI }}0.08,{ }1.9} \right){\text{ Gy}}^{ - 1}$	A correlation of 0.7 was assumed between the logs of $\beta_{1} + 1$ and $\beta_{2} + 1$ to approximate the likelihood, Fig. 2 in Blettner and Boice (1991)
Curtis et al. (1994)	${\text{ERR}} = \beta_{1} d_{{{\text{mean}}}}$	$\beta_{1} = 0.13 \,\left( {95\% \,{\text{CI}} \,{0.04, \,0.27}} \right) \,{\text{Gy}}^{ - 1}$	Both models were assigned a weight of 12.5%
	$1 + {\text{ERR}} = \left[ {1 + \beta_{1} d_{{{\text{mean}}}} } \right]{\text{ exp}}\left( { - \beta_{2} d_{{{\text{mean}}}} } \right)$	$\beta_{1} = 4.7 \,\left( {95\% {\text{ CI }}1.1,{ }13} \right){\text{ Gy}}^{ - 1}$ $\beta_{2} = 0.9 \,\left( {95\% {\text{ CI }}0.35,{ }1.4} \right){\text{ Gy}}^{ - 1}$	
Weiss et al. (1995)	${\text{ERR}}_{l} \left( d \right) = \beta_{1} d \left( {\beta_{2} } \right)^{d} e^{{ - 0.058 \left( {{\text{tse}} - 10} \right)}}$	$\beta_{1} = 12 \,\left( {95\% {\text{ CI }}2.2,{ }52} \right){\text{ Gy}}^{ - 1}$ $\beta_{2} = 0.53 \,\left( {95\% {\text{ CI }}0.21,{ }0.83} \right)$	Here, ${\text{tse}}$ refers to time since exposure [years]. To avoid sporadic samples with huge risk, $\beta_{2}$ was confined in (0.01, 0.99)
Travis et al. (2000)	${\text{ERR}} = 0.1\beta_{1} d_{{{\text{mean}}}}$	$\beta_{1} = 2.7 \,\left( {95\% {\text{ CI }}0.2,{ }12} \right){\text{ Gy}}^{ - 1}$	

The leukaemia high-dose model was obtained by sampling from the different models in this table, each with weight of 25% if not specified differently

For the low-dose model, incidence data in the LSS were grouped into four leukaemia types, and each was modelled separately (Ulanowski et al. 2020). The models showed strong age and dose dependencies. Two slight modifications were applied in the present work. First, since for chronic lymphocytic leukaemia only the male but not the female-specific risk model yielded a radiation risk, we used a mixture of the sex-independent and the female-specific model. Second, multi-model inference was performed as usual based only on goodness of fit to the data without exposure-specific preference for linear models.

Given the many models involved and the strong non-linearities, the selected approach of combining low- and high-dose models for leukaemia was different than that for lung and contralateral breast. The organ dose distribution was split into a low-dose and a high-dose regime. The low-dose model was applied to the low-dose regime, the high-dose model to the high-dose regime, and the results were added. The split point was sampled uniformly in the interval from 0 to 1 Gy. This low split point was chosen due to the strong sensitivity of potentially leukemic cells with regard e.g. to cell killing (Weiss et al. 1995).

**Fig. 6:**
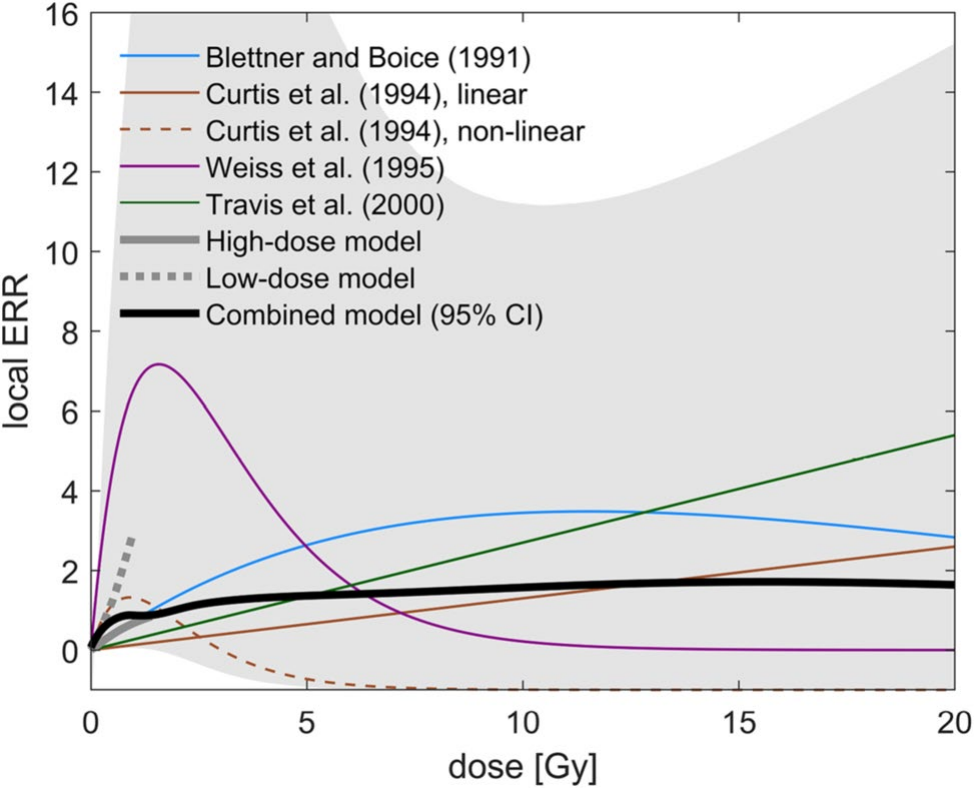
Dose–response curves for leukaemia induction for a 60-year-old woman irradiated at age 50. Coloured lines correspond to the different dose responses entering the high-dose model. Grey lines depict the low- and high-dose model. The low-dose model contributes only up to 1 Gy. The black line corresponds to the combined model, which is identical to the high-dose model above 1 Gy. The grey-shaded region shows the 95% confidence band of the combined model (colour figure online)

The dose–response relationships of the different models over the whole dose range are presented in Fig. 6. The dose–response relationships of the various high-dose models are very different, and even include negative excess risk. The low-dose model is more consistent with the low-dose behaviour of non-linear high-dose models than with linear ones, and shifts the combined model to relatively higher risk at low doses. The combined model features a formidable uncertainty as a result of model selection uncertainty and parameter uncertainties of the individual models.

To give an impression of the contributions of each model, we present in Table 2 the calculated ERRs for our example dose distribution with a mean dose to the red bone marrow of 0.85 Gy and a volume fraction receiving more than 10 Gy (*V*_10 Gy_) of 2%. In determining bone marrow doses, relative bone marrow contents of the different compartments were considered (Simonetto et al. 2019a). Each of the ERRs in Table 2 was obtained by applying one of the high-dose models to the whole dose range of the example dose distribution. Best estimates of the ERR vary from 0.11 to 1.6, and some of the 95% confidence intervals do not overlap. Although the inclusion of the low-dose model increases the risk estimate, the ERR of the combined model is lower than the average of the results from individual high-dose models. The reason is that uncertainty intervals are asymmetric and the reported best estimates refer to the medians of the risk distributions. When the risk distributions of different models are combined, medians are not additive.

**Table 2:** ERRs and 95% CIs for the different high-dose models and for the combined model over the whole dose range, applied to an example dose distribution derived with a standard whole breast 3D-CRT plan, and evaluated for a woman at age 60, irradiated at age 50

Reference	Model type	ERR (95% CI)
Travis et al. (2000)	Linear	0.23 (0.01; 0.94)
Curtis et al. (1994)	Linear	0.11 (0.02; 0.21)
Curtis et al. (1994)	Non-linear	1.3 (− 0.16; 5.6)
Blettner and Boice (1991)	Non-linear	0.22 (0.06; 0.66)
Weiss et al. (1995)	Non-linear	1.6 (0.29; 7.5)
Combined model	Non-linear	0.48 (0.11; 3.5)

### Heart disease model

The heart can compensate some local damage. Furthermore, the organ is remarkably structured, and distinct substructures likely differ in their sensitivity to radiation in terms of finally leading to heart disease mortality. These arguments question the use of Eq. (1) for heart disease mortality following radiation exposure. At present, however, little is known on these issues. Therefore, we based our heart disease model on mean heart dose, the dose metric investigated most often in the literature.

**Fig. 7:**
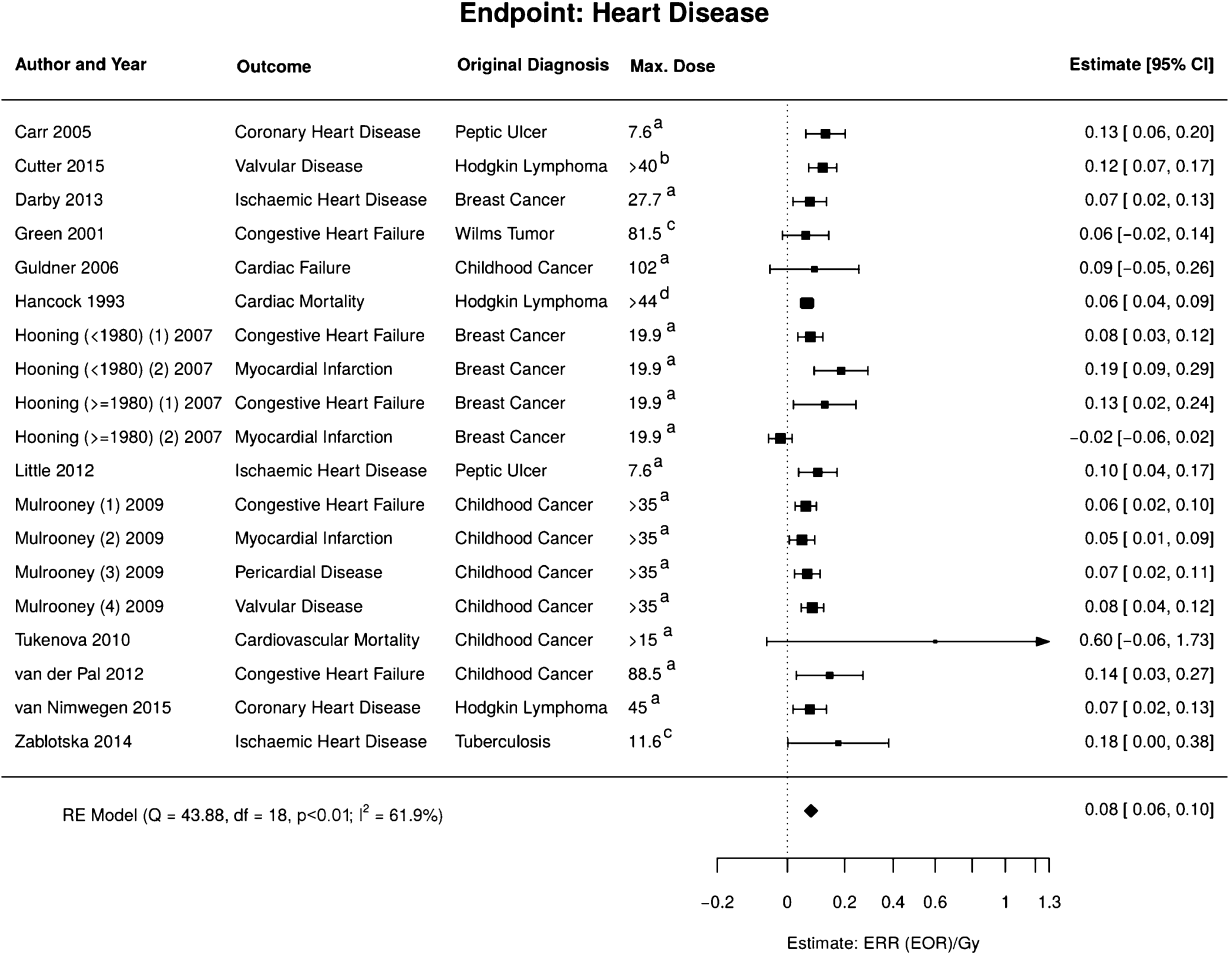
Meta-analysis of the high-dose models for radiation-induced heart disease. *RE model* random effects model, *Q* Cochran’s *Q*, *df*  degrees of freedom, *p* *p* value for test of heterogeneity; I^2^ measure for inconsistency, *ERR* excess relative risk, *EOR*  excess odds ratio. Dose definitions: a mean heart dose; b dose of affected valve; c mean lung dose; d mediastinal dose (colour figure online)

The different studies included in the meta-analysis (Carr et al. 2005; Cutter et al. 2015; Darby et al. 2013; Green et al. 2001; Guldner et al. 2006; Hancock et al. 1993; Hooning et al. 2007; Little et al. 2012; Mulrooney et al. 2009; Tukenova et al. 2010; van der Pal et al. 2012; van Nimwegen et al. 2016; Zablotska et al. 2014) were based on different exposure situations and analysed different endpoints. Nevertheless, most were consistent with a linear dose–response relationship with ${\text{ERR}}_{pd} = 0.08$ (95% CI 0.06, 0.10) Gy^−1^, see Fig. 7.

Parts of the heart may be exposed to low doses. However, as the heart disease model is based on the mean heart dose and not on the local dose distribution, there was effectively no need to combine the high-dose model with a low-dose model. Moreover, the model is consistent with risk estimates from the LSS data (Schöllnberger et al. 2018; Shimizu et al. 2010). Therefore, the model is simply linear in the mean heart dose.

An important quantity for assessing long-term risk is the latency time between exposure and occurrence of radiation-induced heart mortality (Simonetto et al. 2019b). However, this latency time is largely unknown. In particular, there is conflicting evidence from two large studies on heart disease after RT of the breast: in Darby et al. (2013) the ERR was highest within the first 10 years after exposure and lower afterwards. Furthermore, risk was already increased in the first 5 years after exposure. On the other hand, in Henson el al. (2013) a consistent increase in risk was observed with increasing time since exposure, and the risk was highest for more than 20 years after exposure. To reflect both sources of evidence, the ${\text{ERR}}_{pd}$ for heart diseases was multiplied in this work by a latency factor of the form $\Theta + \left( {1 - \Theta } \right) \cdot {\text{tse}}/20$ if the time since exposure tse was less than 20 years. No latency correction was applied for more than 20 years. The parameter Θ represents the unknown fraction of the relative risk that sets in without delay, and was sampled from a uniform distribution in (0, 1). Thus, the mean of the correction factor increases linearly from 0.5 directly after exposure to 1 for 20 years or more after exposure.

### Risks for different sites and comparison to randomised trials

To calculate representative organ-specific risk estimates, we applied the risk models to an example dose distribution of 3D-CRT left-sided breast irradiation, for 10 years after treatment performed at age 50. Mean organ doses and the calculated risks are presented in Table 3 for several organs selected according to organ dose and baseline frequency. Note, however, that some of the risk models do not work with the mean organ doses, but have to be integrated over the actual dose-volume distribution in the given organ. The largest EARs were calculated for the contralateral breast (35 per 10^5^ person years) and lung (20 or 356 per 10^5^ person years for a non-smoker or a heavy smoker, respectively). Among the organs not shown, urinary system cancers were the most relevant for our example exposure, with an EAR of 5.8 per 10^5^ person years.

**Table 3:** Mean organ doses from an example left-sided 3D-CRT plan and calculated excess relative risks (ERR) and excess absolute rates (EAR) for heart disease mortality and cancer incidence

Organ	Present work			Randomised trials	
	Mean organ dose [Gy]	ERR (95% CI)	EAR per 10^5^ person years	Mean organ dose [Gy]	Excess rate ratio (95% CI)
Heart (mortality)	3.2	0.19 (0.12; 0.28)	11 (7.1; 17)	6.3	0.30 (0.15; 0.46)
Contralateral breast	1.0	0.12 (0.05; 0.26)	35 (15; 77)	Not reported	0.20 (0.08; 0.33)
Lung, non-smoker	3.8	0.75 (0.39; 1.2)	20 (10; 32)	9.6	1.10 (0.48; 1.98)
Lung, 25 cigs./day	3.8	0.60 (0.24; 1.0)	356 (138; 641)		
Oesophagus	0.61	0.21 (0.09; 0.56)	1.2 (0.52; 3.2)	8.4	1.42 (0.19; 3.92)
Pancreas	0.53	0.18 (0.08; 0.49)	4.0 (1.7; 11)	Not reported	0.64 (− 0.02; 1.76)
Stomach	0.69	0.32 (0.15; 0.53)	5.3 (2.6; 8.9)	Not reported	− 0.20 (− 0.45; 0.17)
Colon	0.14	0.05 (0.00; 0.22)	2.1 (0.2; 9.5)	Not reported	0.15 (− 0.09; 0.45)
All solid cancer except breast, non-smoker		0.13 (0.09; 0.18)	51 (34; 73)		
Red bone marrow (leukaemia)	0.85	0.48 (0.11; 3.5)	7.8 (1.9; 57)	Not reported	0.71 (0.05; 1.79)
All cancer except breast, non-smoker		0.15 (0.10; 0.27)	60 (40; 111)		0.23 (0.12; 0.36)

Risk calculations were performed for a woman at age 60, treated with breast cancer RT at age 50. For comparison, estimated organ doses and observed excess rate ratios with 95% confidence intervals are replicated from an analysis of randomised trials of breast cancer RT versus no RT (Taylor et al. 2017)

To benchmark the present results, Table 3 also shows rate ratios of heart disease mortality and cancer incidence for breast cancer RT versus no RT, as reported in an analysis of randomised trials (Taylor et al. 2017). For ease of comparison to the ERR, rate ratios were transformed to excess rate ratios by subtraction of 1. It is advantageous to compare relative risks instead of absolute rates, as the latter depend strongly on age and baseline rates. Unfortunately, the organ doses were not reported, apart from a few organs for which retrospective dose assessment was performed. Heart and lung doses were about twice as high in the trials than in the present study, and the dose to the oesophagus was even about tenfold higher. These higher doses can partially be explained by outdated RT techniques, but especially the high dose to the oesophagus resulted from inclusion of radiation fields targeting the internal mammary chain and supraclavicular fossa in many of the randomised trials.

Comparing risks, our estimates for heart diseases, lung cancer and oesophageal cancer were lower compared to the randomised trials, and consistent with the reduction in the organ doses. Regarding other organs, risks were more difficult to compare, as organ doses are unknown for the randomised trials. For most sites, however, risks were of similar size, and uncertainties were large in both approaches. Only for stomach cancer, the observed excess rate ratio was negative, probably due to statistical fluctuations. To cope with the low risk, a modification of our general methodology was applied, transferring the risk for stomach cancer only multiplicatively between the LSS and Germany. Using a mixture between multiplicative and additive transfer as for the other organs, stomach cancer risk would be about 5 times higher than the results in Table 3 because the LSS baseline risk for stomach is about 10 times higher compared to the modern German population.

## Discussion

Dose–response relationships for dose ranges relevant in breast cancer RT were derived by combining evidence from high- and low-dose studies. For low doses, we took advantage of existing detailed, established, and approved risk models, mainly derived from the LSS cohort of the atomic bomb survivors. However, these models do not correctly predict risk from high doses as applied in RT (Berrington de Gonzalez et al. 2013). This is plausible since, compared with low doses, additional cellular mechanisms such as cell killing and repopulation start to play an important role at high doses. Therefore, studies on medical exposures were used to derive risk estimates for the high-dose regime. The different sources of information were fused into joined dose–response curves. Organ cancer risks were evaluated by integrating the dose–response relationship with the organ dose distribution. Biologically, this approach is motivated by the cellular origin of cancer and assumes that long-distance effects are of minor importance for cancer induction. Thus, tumour risk can be inferred from local radiation-induced cellular changes alone. However, it is important to note that this locality assumption does not neglect potential radiation-induced effects of the microenvironment on tumour development. If present, such effects are included in the risk coefficients of the epidemiological and medical studies applied to the low- and high-dose regimes.

Our approach effectively takes into account that biological mechanisms may differ depending on the local dose. Specific mechanistic models for RT-related exposures have been developed, describing cell killing and repopulation (Sachs and Brenner 2005; Shuryak et al. 2009a, 2009b; Schneider 2009). However, these models have to make specific assumptions on the roles of underpinning processes, which may be oversimplified and not testable. Therefore, for the present work we preferred to use a more conservative phenomenological approach by directly accounting for evidence from all available datasets. Nevertheless, mechanistic models may be useful, for example to ensure a reasonable dose–response relationship throughout the parameter space. By use of the local dose in our approach, biological mechanisms may be incorporated in the future.

An essential element in RT applications is the use of fractionation. Modern developments include changes in the fractionation scheme, e.g. in hypofractionated RT the number of fractions is reduced while the dose per fraction is increased. Fractionation is likely important for risk at high doses (Brenner 2012). Using a mechanistic model that includes cell killing and repopulation, Schneider et al. (2010) predicted that the risk of carcinoma induction decreases by about 10% per 1 Gy increase in fraction dose, e.g. by increasing the fraction dose from 2 to 3 Gy. While the result depends on the underlying model structure and parameters, it can provide an estimate of the magnitude of such an effect. In this work, the risk estimates of most of the high-dose studies considered were derived from treatments using traditional fractionation schemes. No epidemiological data exist that could provide estimates of the influence of the fractionation scheme on late health risks. For the low-dose models, the LSS is a cohort with a single, unfractionated exposure. However, fractionation can be expected to be less relevant for risk in the low-dose range. Moreover, the type of exposure of the atomic bomb survivors, which received external exposure with dominantly high-energy photons, applied in a short period of time with high-dose rates, has similarities to RT-type exposures.

For lung and breast cancer, the radiation dose response from the high-dose studies could be well described by a linear relationship (NCRP 2011). Thus, a meta-analysis with linear risk models at high doses was performed. Still, it may be difficult to detect potential non-linearities of the dose response in the high-dose region by epidemiological studies. Due to the strong dose gradients in RT, it is very difficult to estimate the dose at the exact origin of the tumour. Schneider et al. (2018) have shown that dosimetric uncertainty due to tumour size and location alone is sufficient to obscure a potential underlying non-linear dose–response relationship. Although our present models assume linear risk at high doses, it is possible to adjust the dose response for potential emerging deviations from linearity.

The low-dose risk models used for this study fully take into account risk modifications, e.g. by attained age or age at exposure. As derived by the meta-analysis, this is not the case for the high-dose models. This leads to different age dependencies of the low- and high-dose risk models. While this may be difficult to explain biologically, the organ-integrated risk depends on these modifiers in an intermediate way. Using these models for calculations of lifetime risk, the dependence on age is further alleviated by integrating over attained age.

Baseline rates were taken from the general female population. Using the SEER database for second solid cancers after a first breast cancer in the US, it was shown that for most organs the risk of second solid cancer for breast cancer patients without RT is very compatible to the cancer risk for the general female population (Berrington de Gonzalez et al. 2010). However, baseline rates for contralateral breast cancer strongly depend on individual hormonal and genetic risk factors (Lee et al. 2014). Currently, these are not taken into account and may be part of future improvements on personalisation.

For lung cancer, smoking intensity is an important risk factor. The atomic bomb survivors show a high excess relative radiation risk per dose for non-smokers and moderate smokers, and little to no excess relative risk for heavy smokers (Furukawa et al. 2010; Cahoon et al. 2017). Studies after medical radiation exposure clearly show significant radiation risk for smokers, consistent with a multiplicative radiation-smoking interaction (Ford et al. 2003; Kaufman et al. 2008; Neugut et al. 1994; Travis et al. 2002). Therefore, no modification of relative radiation risk with smoking was built into the high-dose model. As a consequence, the combined lung cancer dose–response relationship is either strongly super- or sublinear at low doses, depending on smoking intensity (Fig. 3). Nevertheless, the organ-integrated lung cancer relative risk is not strongly affected by smoking intensity. Of course, baseline rates increase strongly with the number of cigarettes smoked, and therefore, also radiation-induced absolute rates increase as well. Smoking cessation can thus be expected to reduce also radiation-induced absolute rates of lung cancer.

For breast cancer, the low-dose model is based on a pooled breast cancer study that includes not only the LSS, but also several studies with medical exposures (Preston et al. 2002). It depends strongly on age at exposure and additionally on attained age, while the high-dose model is independent of age modifiers. Therefore, the form of the dose–response relationship also depends on the age modifiers, see Fig. 5. Still, there is conflicting evidence about the dependence of breast cancer risk on age at exposure. For the LSS alone, the preferred ERR model was dependent on attained age, but essentially independent on age at exposure after correcting for attained age (Preston et al. 2007; Brenner et al. 2018). Comparing different RT studies, Schneider and Walsh (2015) found a decreasing risk with increasing age at exposure; however, no data were available for persons with age at exposure of 50 years or older. A more detailed discussion on these points can be found in Eidemüller et al. (2021) and the corresponding supplement.

Compared to the same mean bone marrow dose, studies of medically irradiated populations have observed lower leukaemia risk than seen among the Japanese atomic bomb survivors (NCRP 2011). Given the high radiosensitivity of the hematopoietic system, killing or sterilisation of potentially leukemic cells is expected above exposures of about 1 Gy (Weiss et al. 1995). The resulting dose–response relationship is, therefore, very likely non-linear. However, there is no generally accepted form of the dose–response function. In this work, using the combination from the LSS model at low doses and several high-dose models, the resulting dose–response relationship increases relatively strongly at low doses, and is compatible to a plateau at doses above about 1 Gy, albeit with large uncertainties as shown in Fig. 6. To reflect the associated uncertainty, the combined model was obtained by superposition of models from several studies, including linear models.

For heart diseases, a model linear in the mean heart dose has been applied based on overall epidemiological evidence. However, there is no plausible mechanistic basis for the assumed linearity. The heart is a structured organ and each of its component is vital. No robust epidemiological risk estimates from exposures of individual heart substructures exist to date. Due to the heterogeneous dose distribution, potential non-linearities in the dose response may have remained hidden in epidemiological studies (Schneider et al. 2017). Therefore, it is unclear to which extent a reduction of mean heart dose is beneficial if it comes at the cost of higher dose heterogeneity. An improved mechanistic understanding of radiation-induced heart diseases together with advanced modelling and epidemiological data would be highly desirable to better evaluate risks from exposures with different dose distributions.

The presented risk models were implemented in the dedicated software tool PASSOS (2021). For various dose distributions depending on RT technique and individual anatomy (Kundrát et al. 2019b, 2019c), the tool allows to calculate age-integrated risks and average affected/lost lifetime and associated uncertainties (Eidemüller et al. 2019). The PASSOS software takes the patient’s age at RT and other personal factors into account. Although developed primarily for the German population, the proposed methodology can be generalised in a straightforward manner to other populations as well. Further details to the software and examples of clinical applications will be discussed elsewhere.

## Conclusions

Integrating epidemiological evidence from low and high doses, risk models for cancer and heart disease were developed that describe the dose response for dose ranges as applied in breast cancer RT. The models can be used to assess long-term health risk for diverse dose distributions from modern RT techniques. We consider the presented methodology as a flexible framework that allows to estimate risk over a wide dose range. It can be improved in the future with refined epidemiological data and models, additional integration with radiobiological mechanisms, and enhanced personalisation.

## Supplementary Information

Below is the link to the electronic supplementary material.

Supplementary file1 (PDF 544 kb)

## Footnotes

1 Motivated by RT doses of the order of 50 Gy, throughout this paper, we denote doses below a few Gy as ‘low doses’, even if such doses are not considered low from the radiation protection perspective.

2 PASSOS stands for German “Personalisierte Abschätzung von Spätfolgen nach Strahlenexposition und Orientierungshilfe für Strahlenanwendungen in der Medizin”, Personalised assessment of late health risks after exposure to ionising radiation and guidance for radiation applications in medicine.
