# Supplementary material for: Estimating long-term health risks after breast cancer radiotherapy: merging evidence from low and high doses
Source: Radiat Environ Biophys. 2021 Jul 17;60(3):459–74. doi: 10.1007/s00411-021-00924-8 (PMC8310522; doi:10.1007/s00411-021-00924-8)
Supplement: Supplementary file 1 — Supplementary file1 (PDF 544 kb) [file 411_2021_924_MOESM1_ESM.pdf]

Supplementary Material

Estimating long-term health risks after breast cancer radiotherapy: Merging evidence from low and high doses

German baseline rates

Baseline risks were based on German population data (Federal Health Reporting 2016; RKI 2013). The data is provided stratified in 5-year age categories. In order not to underestimate total cancer risk when summing over all organs, incidence and mortality rates of unknown cancer types (ICD-10 codes C76, C80) were redistributed. The observed number of cases of a certain endpoint for a given age category were considered to follow from a Poisson distribution with rate λ. This rate λ may deviate from the observed population rate in particular for very rare diseases or at young ages. Therefore, λ was sampled from the age-specific number of incidence cases. In order to avoid discontinuities, rates were interpolated linearly between age categories.

German baseline rates for lung cancer with smoking

For lung cancer (ICD-10 C33, C34) the baseline rates were personalized according to smoking behaviour. This required separating the population lung cancer risk into the contributions from subgroups of different smoking behaviour. For this purpose, smoking intensity dependent relative risk RR_s was adopted from Pesch et al. (2012), differentiating between the following subgroups: non-smokers, former smokers, <10 cigarettes per day, 10-20 cigarettes per day, 20-30 cigarettes per day, and more than 30 cigarettes per day. Uncertainty in relative risks was sampled from a log-normal distribution based on the published confidence intervals. Age and sex dependent smoking prevalence p_s was taken from Piontek et al. (2016). However, their classification of different smoking intensities misses the highest smoking category present in Pesch et al. (2012). Therefore, it was assumed that 17% of female smokers with more than 20 cigarettes per day actually smoke more than 30 cigarettes a day (Pesch et al. 2012). Moreover, in Piontek et al. (2016) prevalence of smoking is only presented up to an age of 65. For ages above 65, data from Zeiher et al. (2017) was thus used for prevalence of non-smokers, former smokers and smokers, and the shares of subgroups of different smoking intensities were kept from the highest age group in Piontek et al. (2016). Statistical uncertainty was taken into account by sampling the prevalence from the Dirichlet distribution, assuming conservatively 500 cases per age category in the data. Finally, lung cancer rates in non-smokers $\lambda_{non-smoker}$ can be deduced from writing the population lung cancer rate λ as the sum of contributions from the different subgroups in smoking intensity s, each with rate $RR_s \, \lambda_{non-smoker}$:

$$\lambda = \sum_s p_s \, RR_s \, \lambda_{non-smoker}$$

References

Federal Health Reporting (2016) Deaths, Mortality figures (from 1998). Data for year 2014. Joint Service by RKI and Destatis. http://www.gbe-bund.de/. Accessed 2 March 2021

Pesch B et al. (2012) Cigarette smoking and lung cancer--relative risk estimates for the major histological types from a pooled analysis of case-control studies Int J Cancer 131:1210-1219

Piontek D, Atzendorf J, Gomes de Matos E, Kraus L (2016) Kurzbericht Epidemiologischer Suchtsurvey 2015. IFT Institut für Therapieforschung, Munich

RKI (2013) German Centre for Cancer Registry Data. Database Query with estimates for cancer incidence, prevalence and survival in Germany, based on data of the population based cancer registries. Robert Koch Institute. https://www.krebsdaten.de/database. Accessed 2 March 2021

Zeiher J, Kuntz B, Lange C (2017) Rauchen bei Erwachsenen in Deutschland Journal of Health Monitoring 2:59-65 doi:10.17886/RKI-GBE-2017-030
